# Supplementary material for: Relationships between Anxiety, Repetitive Behavior and Parenting Stress: A Comparative Study between Individuals with Autism from Spain and Colombia
Source: Brain Sci. 2024 Sep 9;14(9):910. doi: 10.3390/brainsci14090910 (PMC11430657; doi:10.3390/brainsci14090910)
Supplement: Supplementary file 1 [file brainsci-14-00910-s001.zip › brainsci-3137051-supplementary.pdf]

Supplementary material

Supplementary Figure S1

The polychoric correlation matrix between the RBS-R and the PSI in the Spanish sample of individuals with ASD.

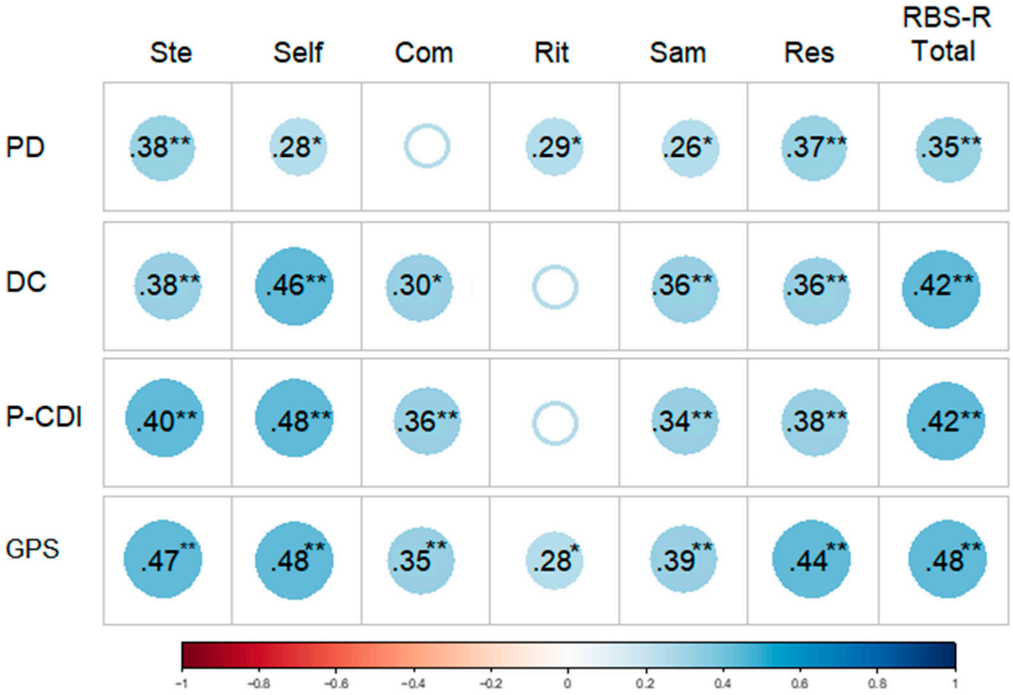

Note: Ste= Stereotypic; Self= Self-Injurious; Com= Compulsive; Rit= Ritualistic; Sam= Sameness; Res= Restrictive Behaviors; PD= Parental Distress; DC= Difficult Child; P-CDI= Parent–Child Dysfunctional Interaction; GPS= Global Parenting Stress;
***=p<.01; *=p<.05*

Supplementary Figure S2

The polychoric correlation matrix between the RBS-R and the PSI in the Colombian sample of individuals with ASD.

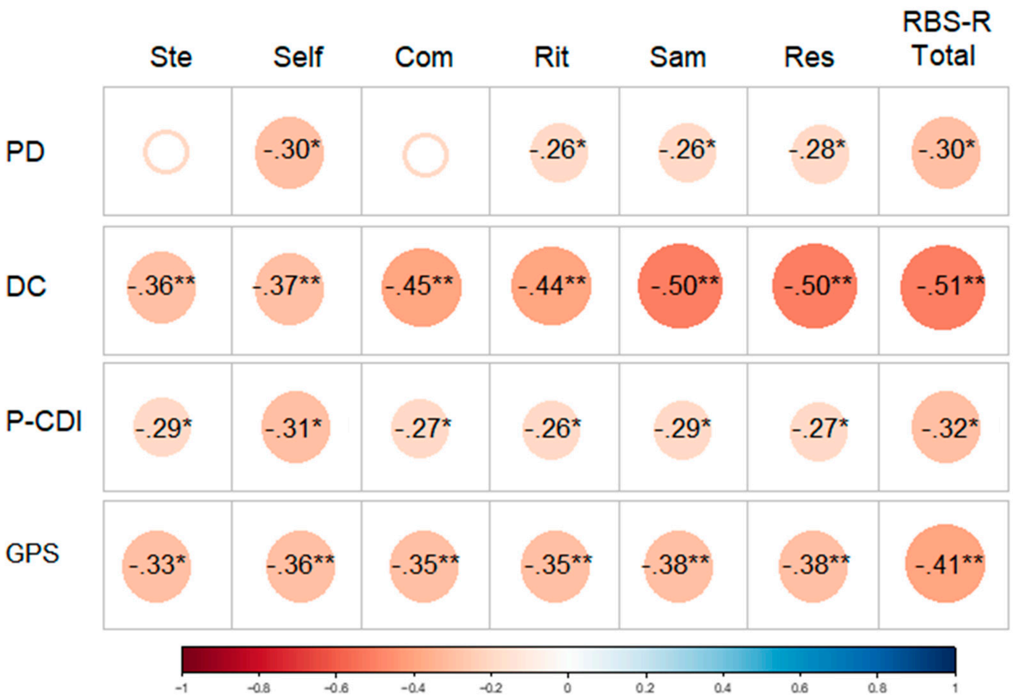

Note: Ste= Stereotypic; Self= Self-Injurious; Com= Compulsive; Rit= Ritualistic; Sam= Sameness; Res= Restrictive Behaviors; PD= Parental Distress; DC= Difficult Child; P-CDI= Parent–Child Dysfunctional Interaction; GPS= Global Parenting Stress;

***=p<.01; *=p<.05*
